# Incidence and Outcome of Acute Phosphate Nephropathy in Iceland

**DOI:** 10.1371/journal.pone.0013484

**Published:** 2010-10-19

**Authors:** Vala Kolbrún Pálmadóttir, Hjalti Gudmundsson, Sverrir Hardarson, Margrét Árnadóttir, Thorvaldur Magnússon, Margrét B. Andrésdóttir

**Affiliations:** 1 Department of Internal Medicine, Landspitali University Hospital, Reykjavik, Iceland; 2 Department of Pathology, Landspitali University Hospital, Reykjavik, Iceland; 3 Division of Nephrology, Landspitali University Hospital, Reykjavik, Iceland; 4 Department of Internal Medicine, Akranes Hospital, Akranes, Iceland; Universidade de Sao Paulo, Brazil

## Abstract

**Background:**

Oral sodium phosphate solutions (OSPS) are widely used for bowel cleansing prior to colonoscopy and other procedures. Cases of renal failure due to acute phosphate nephropathy following OSPS ingestion have been documented in recent years, questioning the safety of OSPS. However, the magnitude of the problem remains unknown.

**Methodology/Principal Findings:**

We conducted a population based, retrospective analysis of medical records and biopsies of all cases of acute phosphate nephropathy that were diagnosed in our country in the period from January 2005 to October 2008. Utilizing the complete official sales figures of OSPS, we calculated the incidence of acute phosphate nephropathy in our country. Fifteen cases of acute phosphate nephropathy were diagnosed per 17,651 sold doses of OSPS (0.085%). Nine (60%) were women and mean age 69 years (range 56–75 years). Thirteen patients had a history of hypertension (87%) all of whom were treated with either ACE-I or ARB and/or diuretics. One patient had underlying DM type I and an active colitis and one patient had no risk factor for the development of acute phosphate nephropathy. Average baseline creatinine was 81.7 µmol/L and 180.1 at the discovery of acute renal failure, mean 4.2 months after OSPS ingestion. No patient had a full recovery of renal function, and at the end of follow-up, 26.6 months after the OSPS ingestion, the average creatinine was 184.2 µmol/L. The average eGFR declined from 73.5 ml/min/1.73 m^2^ at baseline to 37.3 ml/min/1.73 m^2^ at the end of follow-up. One patient reached end-stage renal disease and one patient died with progressive renal failure.

**Conclusion/Significance:**

Acute phosphate nephropathy developed in almost one out of thousand sold doses of OSPS. The consequences for kidney function were detrimental. This information can be used in other populations to estimate the impact of OSPS. Our data suggest that acute phosphate nephropathy may be greatly underreported worldwide.

## Introduction

Acute kidney injury (AKI) and subsequent chronic kidney disease (CKD) have been reported after bowel cleansing with oral sodium phosphate solutions (OSPS) [1]. The clinical pathological entity has been termed “acute phosphate nephropathy” and is caused by deposition of calcium phosphate crystals in the renal tubules, resulting in acute tubular injury and eventual tubular atrophy and interstitial fibrosis [2]. The first biopsy proven case report of this toxic side effect on the kidneys was published in 2003 [3], 13 years after sodium phosphate solutions began to be used as a purgative for colonoscopy [4]. Subsequently, fewer than 40 cases of acute phosphate nephropathy have been reported worldwide, with risk factors of age (60+), female gender, hypertension, angiotensin converting enzyme inhibitors (ACE-I), angiotensin receptor blockade (ARB), diuretics and chronic kidney disease [5]–[7]. During the follow-up of patients with acute phosphate nephropathy, four progressed to end-stage renal disease while the remaining patients manifested chronic kidney disease [7]. Thus, while an association between acute phosphate nephropathy and the ingestion of OSPS has been well documented, there are no clear indications of the global magnitude of this clinical problem. In fact, most clinicians assume that acute phosphate nephropathy is a rare but serious side effect of OSPS. In contrast, we hypothesize that the magnitude of the problem is highly underestimated and in fact that the majority of cases remain unrecognized. Most cases of acute phosphate nephropathy are clinically silent, and are often diagnosed by chance many months after the ingestion of the OSPS. Furthermore, elderly patients with an unexplained rise in creatinine without urinary abnormalities are not likely to undergo a renal biopsy. As OSPS has been a popular purgative for colonoscopy for almost 20 years in many countries it is possible that many individuals have suffered unrecognized acute and subsequent chronic kidney injury following ingestion of OSPS. A better knowledge of the incidence of acute phosphate nephropathy could help to elucidate the magnitude of this clinical problem.

Here we present a population based, retrospective analysis of all cases of acute phosphate nephropathy in Iceland. Furthermore, we are able to provide the minimum incidence rate of acute phosphate nephropathy in our country utilizing the complete official sales figures of OSPS in Iceland.

## Materials and Methods

This is a retrospective analysis of medical records and biopsies of all cases of acute phosphate nephropathy that were diagnosed at Landspitali University Hospital in Reykjavik, during the period from January 2005 to October 2008. The study was approved by the Icelandic Data Protection Authority, the Bioethics Committee of Landspitali University Hospital and written consent was obtained from all living participants.

Diagnostic criteria were, as proposed by Markowitz et al. [8], (1) (acute) renal failure (2) pathologic findings of acute and/or chronic tubular injury with abundant tubular calcium phosphate deposition and (3) exposure to oral sodium phosphate bowel cleansing (Phosphoral (45 ml×2), Laboratories Casen-Fleet S.L.U.). Index cases were found by reviewing the pathological diagnosis of renal biopsies performed in the study period. Medical records were reviewed for age, gender, medical history, medication, the ingested dose of OSPS, indication for colonoscopy and symptoms after OSPS ingestion. The following serum creatinine values were documented: at baseline, at presentation of kidney injury, at the time of renal biopsy, the highest available, and the latest value. Estimated glomerular filtration rate (eGFR) was calculated according to the CKD-EPI (Chronic Kidney Disease Epidemiology Collaboration) equation [9]. Other laboratory results that were registered included haemoglobin level, s-calcium, s-phosphate and urinalysis, all were collected at or around the time of detection of renal failure. Patients were followed until September 30^th^ 2009, date of starting renal replacement therapy or death, whichever came first.

Oral sodium phosphate solution (Phosphoral, 45 ml×2) was available without prescription in Iceland during the period March 1999 until May 2009, but since that time it has only been available by prescription. Yearly sales figures of Phosporal (45 ml×2) in Iceland were obtained from the Icelandic Medicines Control Agency (personal communication).

All renal biopsies were obtained and processed at Landspitali University Hospital in Reykjavík. Standard processing of renal biopsies included light microscopy and immunofluorescence. Light microscopy biopsies were stained with hematoxylin and eosin, periodic acid-Schiff, Massons's trichrome, and Jones methenamine silver. All cases were examined under polarized light and stained with von Kossa to differentiate calcium phosphate (nonpolarizable, von Kossa positive) from calcium oxalate (polarizable, von Kossa negative). For immunofluorescence, 3 µm cryostat sections sections were stained with polyclonal FITC-conjugated antibodies to IgG, IgA, IgM, C3, kappa, lambda, fibrinogen and albuminn (Dako). Calcium phosphate deposits were quantified on a scale from 1+ to 4+.

### Statistical analysis

Descriptive summary statistics, including mean, median, standard deviation, and/or range were computed.

## Results

### Incidence rate

Fifteen cases of acute phosphate nephropathy were diagnosed between January 2005 and October 2008. They were preceded by OSPS (Phosphoral) bowel cleansing for colonoscopy in 14 cases and for preparation for colon operation in one. During this period, 17,651 doses of Phosphoral were sold in the country (incidence 0.085%).

### Clinical characteristics of patients

The demographic data and clinical characteristics of the patients are shown in table 1. Sixty percent of the patients were women and the mean age at OSPS ingestion was 68.8 years (SD 7.0, range 56–77 years). Thirteen patients had a history of hypertension and all were treated with either ACE-I or ARB (N = 10) and/or diuretics (N = 8) at the time of OSPS bowel cleansing. One patient had DM type 1 and two patients had DM type 2. Two patients had a history of colitis ulcerosa. One patient had no concomitant illness (patient no 11). None of the patients had known hyperparathyroidism, heart failure or liver disease. At the presentation of kidney disease, 12 patients had s-calcium and phosphate levels within normal range, whereas three patients had low s-calcium and high s-phosphate. The majority of patients (73%) were anaemic at the time of presentation.

**Table 1:** Demographics and clinical characteristics of patients with acute phosphate nephropathy after OSPS ingestion.^a^

Patient	Age, sex	HT	DM	ACE-i/ARB	Diuretics	Proteinuria	Urinary sediment	S- calcium	S-phosphorus	Hemoglobin
1	59F	Y	N	Y	Y	Negative	10–25 wbc, 2–5 rbc	2.38	1.43	108
2	58M	Y	N	Y	Y	260 mg/24 hr	No abnormalities	2.25	1.17	109
3	75F	Y	N	Y	Y	0.13 g/24 hr	No abnormalities	1.05^b^	1.86	112
4	65M	N	Y type 1	N	N	Negative	2–5 wbc, 1–2 rbc^c^	1.97	1.66	126
5	71F	Y	N	Y	N	Negative	5–10 wbc, 1–2 rbc	1.32	1.19	84
6	74F	Y	N	Y	N	Negative	>100 wbc, 1–2 rbc, +++bacteria^d^	1.23	1.60	131
7	69F	Y	N	Y	N	Negative	5–10 wbc, 1–2 rbc	2.20	1.33	114
8	77M	Y	Y type 2	N	Y	Negative	No abnormalities	1.0^b^	2.31	111
9	56M	Y	N	Y	Y	Negative	No abnormalities	2.26	1.12	118
10	74F	Y	N	N	Y	Negative	10–25 wbc, 1–2 rbc	1.21^b^	1.33	114
11	62F	N	N	N	N	Negative	No abnormalities	1.30	1.02	150
12	71M	Y	Y type 2	Y	N	(+)	2–5 wbc, 5–10 rbc c	1.13^b^	0.99	135
13	71M	Y	N	Y	N	Negative	1–2 wbc	1.23^b^	1.41	116
14	75F	Y	N	N	Y	Negative	1–2 wbc, 2–5 rbc^c^	1.23^b^	1.21	85
15	75F	Y	N	Y	Y	Negative	25–50 wbc	1.25^b^	1.39	105

a Abbreviation used in table: HT, hypertension; DM, diabetes mellitus; ACE-i, Angiotensin convertive enzyme inhibitor; ARB, angiotensin receptor blockade; Y, yes; N, no; WNR, withing normal range; wbc, white blood cells; rbc, red blood cells. Normal values for s-calcium, 2.15–2.60 mmol/L; ionized calcium, 1.13–1.33 mmol/L; s-phosphorus, 0.75–1.65 mmol/L; Hemoglobin, 118–152 g/L (women), 134–171 g/L (men).

b Ionized s-calcium.

c In these sediments, hyaline, tubular cell, granular or Muddy Brown casts were also seen.

d After treatment of the UTI, urine analysis was normal.

The indication for colonoscopy was anaemia in four cases, gastrointestinal discomfort (constipation, abdominal pain, diarrhea) in three, unknown in four patients and one each had unexplained weight loss, regular control of colitis ulcerosa (not active at the time of colonoscopy), control of incidental tumor finding on CT and preparation for colon operation. It was documented that all patients had used OSPS for bowel cleansing; four patients took 45 mL bottles ×2 and reported having definitely followed instructions. Patient no 3 threw up the first bottle so she ingested an extra bottle. Patient no 6 had bowel cleansing three times with OSPS in a three month period, two times for colonoscopy and once in preparation for radiography of the colon. The remaining nine patients all ingested OSPS for bowel cleansing, but it is not documented whether they followed the instructions closely or not. Most patients either denied any symptoms following the OSPS ingestion or symptoms were vague and non-specific (no symptoms (5), thirst (2), increased diuresis (1), persistent diarrhea and weight loss (1), malaise (1), cough (1), unknown (4)). One patient (no 4) had colonoscopy because of diarrhoea and was diagnosed with Clostridia difficile colitis. This patient was, unlike the other patients, hospitalized after the colonoscopy and more acutely ill.

### Kidney function

An overview of kidney function before and after ingestion of OSPS is shown for each patient in table 2. Baseline creatinine was available in all patients at the average 4.4 months (SD 6.7 range 0–25.8 months) before the OSPS ingestion. Mean baseline creatinine was 81.7 µmol/L and mean eGFR was 73.5 ml/min/1.73 m^2^. Four patients had an eGFR of <60 ml/min/1.73 m^2^ at baseline. Deterioration of kidney function was diagnosed 4.2 months (mean, SD 4.5, range 1 day–16.3 months) after OSPS ingestion. The average creatinine at discovery of acute kidney injury was 180.1 µmol/L. The peak creatinine was on average 247.2 µmol/L and 217.8 when the biopsy was performed, which occurred at a median 6.2 months (range 0.4–39.8 months) after the ingestion of OSPS. After the initial rise in serum creatinine, it decreased with time in most patients (87%), but progressed in two, one of them received treatment for ESRD (no 8) and one died 17.8 months after OSPS ingestion from pneumonia (no 4). Patients have been followed for an average of 26.6 months after the OSPS ingestion, ranging from 5.3 to 65.9 months and at latest follow-up, the average creatinine was 184.3 µmol/L. The average eGFR declined from 73.5 ml/min/1.73 m^2^ at baseline to 37.3 ml/min/1.73 m^2^ at the end of follow-up and 13 patients had eGFR lower than 60 ml/min/1.73 m^2^.

**Table 2:** Changes in kidney function before and after ingestion of oral sodium phosphate solutions.^a^

Patient	Age, sex	Kidney function -1^b^		Time -1^f^	Kidney function 1^c^	Time 1^g^	Kidney function 2^d^	Time 2^h^	Kidney function 3^e^		Time 3^i^
		SCr	eGFR	Days	SCr	Days	SCr	Months	SCr	eGFR	Months
1	59F	48	103	22	105	288	105	11.7	80	70	20.5
2	58M	67	101	93	170	95	166	9.6	116	59	22.9
3	75F	54	89	253	255	1	380	0.4	173	25	39.2
4	65M	73	93	61	120	0	312	1.0	440	11	17.8
5	71F	62	87	199	153	52	138	4.3	112	43	16.9
6	74F	66	79	10	310	104	308	3.5	121	38	7.7
7	69F	68	79	58	160	146	115	15.1	134	35	33.6
8	77M	92	69	7	131	2	555	39.8	554	8	46.4
9	56M	103	70	92	164	31	125	4.2	114	62	5.3
10	74F	81	62	775	194	45	198	6.2	170	25	27.2
11	62F	88	61	28	127	489	105	22.2	101	51	36
12	71M	110	58	3	144	275	132	18.3	144	41	33.8
13	71M	115	55	315	280	101	290	4.1	208	26	43.1
14	75F	98	49	0	239	185	221	6.6	194	21	23.1
15	75F	100	48	61	150	62	117	3.8	103	45	25.8

a Abbreviations used in table: SCr, s-creatinine in µmol/L; eGFR, estimated glomerular filtration rate in ml/min/1.73 m^2^, calculated using CKD-EPI (Chronic Kidney Disease Epidemiology Collaboration) equation (see Methods).

b Kidney function -1, baseline value of SCr and eGFR (before ingestion of OSPS);

c Kidney function 1, SCr at presentation;

d Kidney function 2, SCr at the time of renal biopsy;

e Kidney function 3, SCr and eGFR at the time of last follow-up.

f Time -1, days between measurement of baseline creatinine and the ingestion of OSPS;

g Time 1, days between the ingestion of OSPS and the discovery of AKI;

h Time 2, months between the ingestion of OSPS and kidney biopsy;

i Time 3, months between ingestion of OSPS and the last follow-up.

The most frequent finding in the urinary sediment was sterile pyuria, with rare red blood cells. In some, hyaline, tubular cell or granular casts were seen. One patient had a urinary tract infection at the time of diagnosis. Urine dipstick was negative for protein in 12 patients and trace in one. Two patients had 24-hr urine protein measured, 260 and 130 mg/day, respectively (Table 1).

### Renal biopsy findings

**Figure 1:**
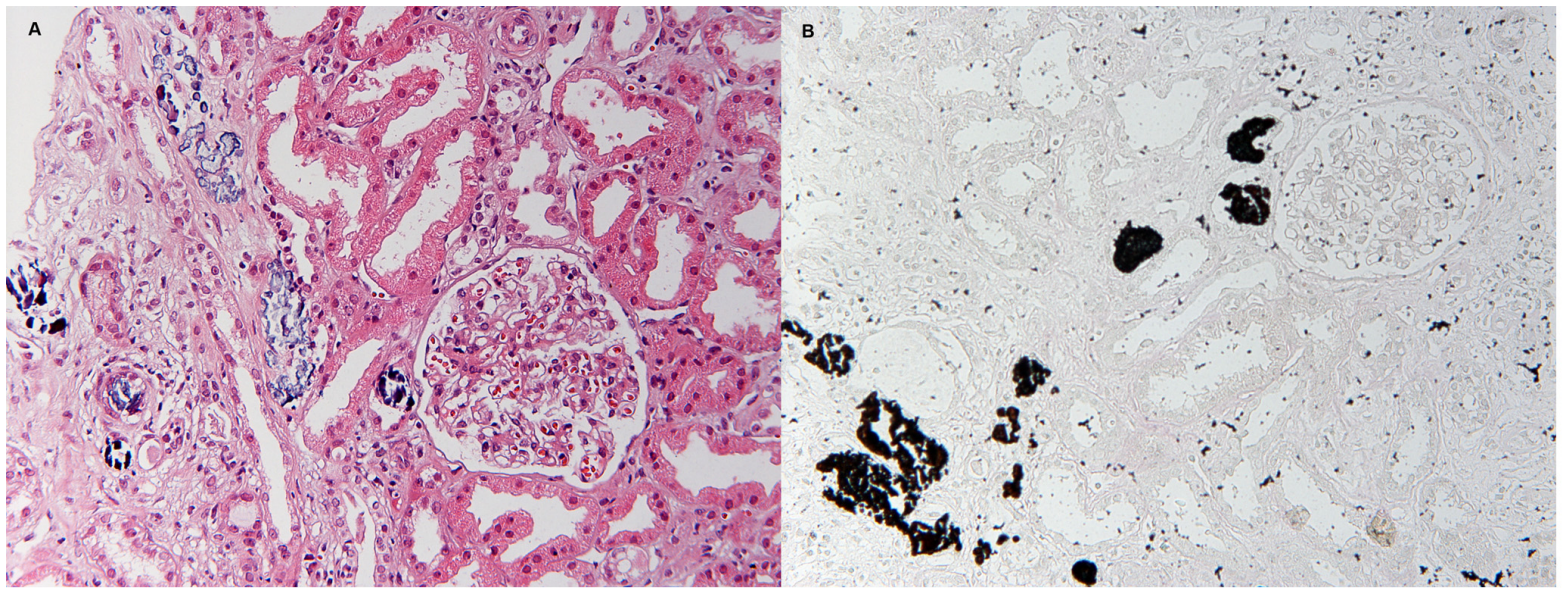
Renal biopsy findings in acute phosphate nephropathy. A) Abundant calcifications are seen within tubules and in the interstitium. Adjacent tubules are athrophic and there is interstitial fibrosis (Hematoxylin and eosin staining, original magnification x 400). B) Positive von Kossa staining in the same biopsy confirms that the calcifications are composed of calcium phosphate (original magnification x 400).

The renal biopsy findings in patients with acute phosphate nephropathy are shown in Table 3. As could be expected from this cohort of elderly people, global sclerosis of the glomeruli was seen in most biopsies (73%). Sampling for light microscopy included a mean of 20.2 glomeruli (range 5–44), and a mean of 3.7 (18.3%) glomeruli were sclerotic. Other glomeruli appeared normal. Vascular disease was mild or absent in most patient and considered to be moderate in one. The typical findings of acute phosphate nephropathy were present in all patients as shown in Figure 1. Calcium phosphate deposits were found mostly in distal tubules and collecting ducts, located within the cytoplasm of tubular epithelial cells, within tubular lumina, or in the interstitium. The calcifications did not polarize and stained intensely with von Kossa stain, which confirmed their composition as calcium phosphate. The quantity of calcium phosphate deposits was not associated with time after OSPS ingestion, increase in creatinine or the final outcome. Tubular injury was accompanied by interstitial oedema in particular in biopsies of two patients who had a biopsy taken at the earliest interval from OSPS ingestion, 12 and 30 days, respectively. Interstitial inflammation was seen in most biopsies (80%), but there did not seem to be a relationship between the length of time from OSPS ingestion to biopsy and the quantity of inflammation. Chronic, irreversible tubular injury in the form of tubular atrophy and interstitial fibrosis was seen in all biopsies, ranging from 10 to 60% of the cortical area sampled.

**Table 3:** Biopsy results of 15 patients after ingestion of oral sodium phosphate solution.^a^

Patient	Glomeruli		Interstitium				Arteriolar hyalinosis	Other pathology
	Total number	Global sclerosis	Ca/P	TA/IF	Oedema	Inflammation		
1	32	2	4+	50%	-	++	none/mild	No
2	29	2	3+	40%	-	±	none/mild	No
3	5	0	3+	30%	++	-	none/mild	No
4	20	0	2+	30%	++	+	mild	No
5	24	4	3+	30%	-	+	none/mild	No
6	22	5	3+	30%	+	+	none/mild	No
7	22	9	2+	40%	-	+	mild	No
8	19	3	2+	60%	-	±	moderate	No
9	22	5	2+	40%	-	±	mild	No
10	10	0	3+	30%	-	±	none/mild	No
11	11	3	3+	10%	-	-	none/mild	No
12	10	4	1+	30%	+	+	mild	No
13	8	0	3+	50%	(+)	-	none/mild	No
14	25	5	3+	30%	-	+	mild	No
15	44	14	3+	50%	-	++	mild	No

a Abbreviations used in table: Ca, calcium; P, phosphate; TA, tubular atrophy; IF, interstitial fibrosis.

## Discussion

Acute phosphate nephropathy is an iatrogenic cause of kidney injury after bowel cleansing with OSPS. The relationship between ingestion of OSPS and the deposition of calcium and phosphate in the tubules, causing injury with subsequent interstitial scarring with concomitant loss of kidney function has been documented in 37 cases since 2003 [7]. The majority of the cases (21) come from a single-center study in the U.S. [2], single cases have been reported from other U.S. centers [3], [10]–[12], Norway [13], Holland [14], Belgium [15], UK [16], Lebanon [17], Korea [18], New Zealand [19], and five from Israel [20], reflecting the widespread use of OSPS. We have diagnosed 15 additional patients with acute phosphate nephropathy since 2005, which are described in detail in this paper. Iceland has a population of 315,000 and one nephrology referral center which offers a unique opportunity to estimate the incidence of acute phosphate nephropathy following OSPS. Comparable estimation has not been possible to do in other countries [21]. Epidemiological studies, undertaken to assess the incidence of acute kidney injury after OSPS, have yielded conflicting results and have not fully elucidated this issue [22]. The largest study comprised 9799 individuals, 50 years of age or older, who had received either OSPS or polyethylene glycol (PEG) for bowel cleansing for colonoscopy. The incidence of acute kidney injury, defined by a ≥50% increase in serum creatinine, was 1.29% and 0.92%, for OSPS and PEG, respectively [23]. These studies are all hampered by the lack of renal biopsies for the diagnosis of acute phosphate nephropathy.

In total, 17,651 doses of Phosphoral (45 ml×2) were sold in Iceland during the study period. The incidence of acute phosphate nephropathy is thus 1/1177 doses of OSPS or 1/2064 if all doses that have ever been sold in the country are taken into account (30,958). However, these numbers likely underestimate the magnitude of the problem, as sold doses of OSPS have not necessarily all been ingested and also because the diagnosis requires a kidney biopsy. Our study shows that the symptoms are frequently vague or even absent, and that the diagnosis of acute kidney injury is commonly found by chance. When these patients present with a rise in creatinine they are unlikely to undergo a renal biopsy for definite diagnosis for several reasons: Individuals at risk for developing acute phosphate nephropathy are elderly people (all above 55 years in our study), have a history of hypertension and are treated with medications that can cause fluctuations in the kidney function, i.e. diuretics and ACE-I or ARB and present with a bland urine sediment without clinically important proteinuria. The lag time between the marketing of the OSPS and the recognition of acute phosphate nephropathy as a side effect, which was six years in our country and up to 13 years in the U.S., emphasizes how latent and unexpected this condition is.

How do our results then compare with findings in other populations? Twenty-five cases of acute phosphate nephropathy have been reported in the medical literature coming from the U.S. [2], [3], [10]–[12]. However, based on our data we would expect 15,000 cases, in light of the proportional size differences of the U.S. and Icelandic populations (the U.S. has approximately thousand fold more inhabitants than Iceland). This striking difference is an obvious argument for the underreporting of acute phosphate nephropathy worldwide. Furthermore, we predict that the prevalence of acute phosphate nephropathy is even greater in the U.S., and the direct extrapolation based on number of inhabitants may be a bias towards an underestimation of the problem. OSPS has been the preferred purgative in both countries; one U.S. center reported sodium phosphate purgatives to be used in up to 80–90% of patients for bowel preparation for colonoscopy [6]. It is, however, likely that colonoscopies are done more frequently in the U.S. compared to Iceland as routine screening for colon and rectal cancer with colonoscopy has not been put into practice in Iceland. In the U.S. the rate of screening colonoscopy increased dramatically after the introduction of full Medicare coverage of the procedure in 2002 [24], [25].

The main limitation of the present study is its retrospective character and the lack of a control group. Our calculation of the national incidence of acute phosphate nephropathy is therefore crude, and most likely biased towards underestimation of the problem. Also there is incomplete information on the adherence to the prescribed hydration regimens and limited ability to find new risk factors. Despite these limitations, this is the only study available that is able to estimate the magnitude of the renal complication of OSPS use.

The large number of cases that have been diagnosed in Iceland compared to other countries is most likely due to the close collaboration of a small group of nephrologists (7) who receive all nephrology referrals in the country. They have been aware of acute phosphate nephropathy as a complication of OSPS use from the beginning of 2005. The nephrologists attend the meetings with a single pathologist and discuss all cases. Such setup can increase the awareness of both sides and may have lead to an increase in biopsies taken, especially when there was a history of exposure of OSPS (which has become a routine question in the case of an unexplained rise in s-creatinine). The pathologist may also have been more observant, especially when the clinician informs about OSPS use, although the diagnosis is rather straightforward and requires abundant tubular deposits of calcium phosphate (Figure 1). Alternatively, the Icelandic population may be more prone to develop acute phosphate nephropathy due to unknown genetic and environmental factors, but remarkable similarities between our patient group and the previously reported cases argues against that. The majority of our cases were women (60%) with an average age of 68.8 years and all patients were older than 55 years. This is comparable to the cases that have been diagnosed worldwide, 81% is female and their mean age was 66.1, ranging from 39–85 years and 70% older than 60 years [2], [3], [10]–[20]. The co-morbidities in our patients were also similar to the other cases, 87% of our patients had hypertension and all used either diuretics, and/or ACE-I or ARB, 20% had diabetes and 27% had e-GFR <60 ml/min per 1.73 m^2^ at baseline. These risk factors have been reported with a similar rate before, 81% of previously reported cases have hypertension and most have been treated with the aforementioned drugs, 14% have diabetes and 22% have baseline e-GFR <60 ml/min per 1.73 m^2^ [7]. In addition to this, inappropriate use of OSPS could be responsible for the high rate of cases in our country. The use of OSPS can be criticized in two of the patients, one who was exposed to repeated doses in a short period of time, and another who turned out to have an active colitis. We included all cases in this study, as clinical practice is clearly not always according to recommendations.

The consequences of acute phosphate nephropathy following OSPS on kidney function and future health of these patients is of major concern. Although the kidney function improved in most patients with time, one patient reached end-stage renal disease and one died with a pre-end-stage renal failure. At the end of follow-up, the eGFR had decreased by an average of 36 ml/min/1.73 m^2^ from the baseline value. Studies have shown that acute kidney injury and chronic kidney disease are associated with an increased risk of cardiovascular disease and mortality [26]–[28].

This study is an important addition to the reported cases of acute phosphate nephropathy and confirms the risk factors that have been suggested in other studies. We were able to reveal the incidence of this complication due to the unique setting in our country. Approximately one out of every thousand sold doses of OSPS resulted in acute kidney injury and follow-up showed that the long-term consequences were not benign. This information can be used in other populations to estimate the impact of OSPS use. Our results suggest that acute phosphate nephropathy following OSPS is underestimated worldwide and that it may be an important iatrogenic cause of chronic kidney disease in the elderly now and in the near future.
